# Integrated care of diabetes during pregnancy: a Qatari nationwide cohort

**DOI:** 10.1016/j.eclinm.2024.102605

**Published:** 2024-04-22

**Authors:** Mohammed Bashir, Ibrahim Ibrahim, Stephen Beer, Hessa Shahbic, Faten Eltaher, Kholoud Al-Mutawaa, Mahmoud Zirie, Abdul-Badi Abou-Samra

**Affiliations:** a Endocrine Section, Internal Medicine Department, Hamad Medical Corporation, P.O Box 3050, Doha, Qatar; b Qatar Metabolic Institute, Hamad Medical Corporation, PO Box 3050, Doha, Qatar; c Sidra Medicine, Qatar Foundation, P.O Box 26999, Doha, Qatar; d Women's Health Program, Primary Health Care Corporation, Box: 26555, Doha, Qatar; e Department of Obstetrics and Gynaecology, Women's Wellness and Research Centre, Hamad Medical Corporation, P.O Box 3050, Doha, Qatar; f Non-communicable Diseases, Public Health Department, Ministry of Public Health, P.O Box. 7744, Doha, Qatar; g Qatar National Diabetes Committee, Ministry of Public Health, P.O Box. 7744, Doha, Qatar

**Keywords:** Universal screening, Diabetes, Prevalence, Pregnancy

## Abstract

**Background:**

Diabetes in pregnancy (DIP) is associated with adverse fetal and maternal outcomes. DIP is classified as either pre-existing or new-onset diabetes mellitus (DM), which is classified into gestational DM (GDM) and newly detected type 2 (N-T2D). All pregnant women in Qatar who are not known to have pre-existing DM are offered screening for DIP during the first antenatal care visit and after 24 weeks gestation. The study aims to report the DIP screening rates, the prevalence of DIP, and the impact of the universal screening program on adverse pregnancy outcomes.

**Methods:**

This retrospective study included all women who gave birth in Hamad Medical Corporation (HMC) hospitals between 2019 and 2022. New-onset DIP was defined using the WHO-2013 criteria. The primary outcomes were the screening rates and the prevalence of DIP in Qatar. The secondary outcomes were the difference in preterm delivery, C-section, macrosomia, large for gestational age (LGA), small for gestational age (SGA), and intra-uterine fetal death (IUFD) between women with or without GDM.

**Findings:**

We included 94,422 women who gave birth to 96,017 neonates (85.7%) out of 112,080 neonates born nationwide. The number of women with pre-existing diabetes was 2496 women. Of 91,926 eligible women, 77,372 (84.2%) were screened for DIP. The prevalence of GDM is 31.6% (95% CI: 31.3–32.0%); N-T2D is 2.2% (95% CI: 2.1–2.3%), and pre-existing Type 2 DM and Type 1 DM was 2.6% (95% CI: 0.8–3.0%) and 0.2% (0.19–0.25), respectively. Compared to the non-GDM group, women with GDM were older (30.8 ± 5.3 versus 29.7 ± 5.2 years, p < 0.001). After adjusting for age, women with GDM had lower risk of IUFD and SGA (0.63 [95% CI 0.50–0.80, p < 0.001], 0.88 [95% CI 0.84–0.92, p < 0.001] respectively) but higher risk of C-section and LFD (1.07 [95% CI 1.04–1.10, p < 0.001], 1.09 [95% CI 1.01–1.15, p = 0.01], respectively, compared to women with no-GDM.

**Interpretation:**

Of the women eligible for screening, 84.2% were screened by the DIP program in Qatar. The prevalence of DIP in Qatar is 36.9%. Integrated care is critical for the screening and management of diabetes during pregnancy.

**Fundings:**

The authors did not receive any funding for this project.

**Research in context**

**Evidence before this study**

Newly detected diabetes in pregnancy (DIP) is associated with serious short and long-term complications. While there is an agreement on the role of screening for DIP, the issue of whom to screen is still debatable. Some centres screen women based on risk stratification, while others screen all women. There is little evidence to support either approach. We performed an extensive literature review on the application of universal screening for universal screening for DIP.

**Added value of this study**

As universal screening is undertaken in Qatar, in this study, we report on the uptake for screening rates for DIP, the prevalence of DIP and the outcomes of severe pregnancy outcomes among women with and without GDM. We show that with integrated care, 85.7% of women who delivered in Qatar were screened for DIP and that 36.6% of the women have DIP. We also show that the adverse severe pregnancy outcomes in women with GDM approximates those in women with no GDM.

**Implications of all the available evidence**

This study shows that integrated care providing screening and managing women with DIP can be successfully implemented. Integrated care between primary and secondary care is critical for the success of such programs.

## Introduction

Diabetes in pregnancy is classified as pre-existing and newly detected diabetes mellitus (DM).^1^ Pre-existing DM affects 0.2–0.3% of all pregnancies and is defined as any form of hyperglycaemia, usually type 1 diabetes mellitus (T1D) and type 2 diabetes mellitus (T2D), known before pregnancy. Newly detected DM affects 14% of all pregnancies and is further classified into gestational diabetes mellitus (GDM) or overt newly detected T2D (N-T2D).^2^ All four forms of diabetes in pregnancy (DIP) are associated with an increased risk of maternal and fetal morbidity and mortality.^3^ Furthermore, offspring born to mothers with diabetes in pregnancy are at higher risk of obesity during childhood and early onset T2D.^4^ Indeed, exposure to higher levels of hyperglycaemia in utero is independently associated with insulin resistance and adiposity during childhood.^5^^,^^6^

Universal screening for diabetes in pregnancy is a point of contention between different international guidelines. Guidelines are divided between universal and selective screening based on risk factors.^7^, ^8^, ^9^ The main concern about universal screening is the increase in the prevalence of GDM among low-risk women.^10^ However, the selective approach is more complex, modestly reduces the screening rates by 10–30%, and misses 33% of cases of GDM.^11^, ^12^, ^13^, ^14^, ^15^ Furthermore, Cosson et al. showed that switching from selective to universal screening was associated with reduced large for gestational age (LGA) rates, preterm delivery, neonatal jaundice, and fetal hospitalisation.^16^ Finally, universal screening was cost-effective and associated with improved quality of life compared to selective or no screening.^17^^,^^18^

Another controversial topic is the gestational age for screening for DIP. The Endocrine Society recommends screening for DIP before 13 weeks gestation using fasting blood glucose (FBG), HBA1c, or random glucose.^19^ The Australian guidelines recommend early screening in women with metabolic risk factors.^20^ However, other societies do not recommend screening before 24 weeks gestation.^9^ There is currently no substantial evidence to support both approaches.^21^ A recent randomised clinical trial (RCT) showed that treatment of early detected GDM was associated with a modest, nonetheless significantly lower risk of adverse pregnancy outcomes compared to no treatment.^22^ The study also showed that repeat screening confirmed GDM in two-thirds of the women in the no-treatment arm.^22^ Early screening aims to uncover undiagnosed pre-existing T2DM. Hence, early screening is justifiable in countries with a high prevalence of T2D.

Qatar is a multicultural country in the Middle East with a high prevalence of obesity and T2D.^23^^,^^24^ Only two previous studies have reported on the prevalence of DIP in Qatar. In 2011, Bener et al. invited women attending the largest tertiary maternity centre in Qatar, the Women's Wellness and Research Centre (WWRC), to undergo screening for DIP and reported a GDM prevalence of 16.3%.^25^ Bashir et al. reported a GDM prevalence of 21.5% following the adoption of universal screening for DIP at 24 weeks gestation.^26^ Since 2017, Qatar has developed the National Diabetes in Pregnancy guideline that commended universal screening for DIP.^27^

This study aims to report the national screening rates for DIP, the prevalence of pre-existing and newly detected DM, and the impact of the national screening on serious pregnancy complications.

## Methods

### Study design and participants

This retrospective study included all women who gave birth in the Hamad Medical Corporation maternity hospitals during the four calendar years between 2019 and 2022 following the implementation of the National Diabetes in Pregnancy guideline. Hamad Medical Corporation (HMC) is Qatar's largest secondary and tertiary care provider and oversees most of the country's deliveries. The study's main objectives are to define the current national screening rate for diabetes in pregnancy, define the prevalence of diabetes in pregnancy, and assess the impact of the national screening program on key pregnancy outcomes among women with or without GDM. We included all women who gave birth in all HMC facilities after 24 weeks gestation between 2019 and 2022. We did not exclude any woman from the study.

### Ethics

The study was approved by the Institutional Review Board in Hamad Medical Corporation (Ref number: MRC-01-21-306). Due to the retrospective nature of the study, no consent was required. The study was conducted in accordance with Helsinki's declaration.

### Procedures

Qatar has two public healthcare providers: primary healthcare is delivered by the Primary Health Care Corporation (PHCC), while secondary and tertiary healthcare is delivered by Hamad Medical Corporation (HMC). All residents in Qatar have access to these services. Both healthcare organisations use a shared electronic medical records system—Cerner. PHCC provides antenatal care through dedicated ANC clinics at the start of pregnancy. HMC has four maternity hospitals that manage most of the deliveries in Qatar. Qatar has developed a specific diabetes-in-pregnancy program as outlined in the National Guidelines.^27^ The program included establishing a national screening program for DIP, a unified management protocol, and a pathway for referral between primary and secondary care. Each primary health care centre has Dietitians and Diabetes Educators to assist in managing GDM. Besides, in each maternity hospital, a dedicated team of Endocrinologists, Obstetricians, Dietetics, Diabetes Nurse specialists, and Nurses was established to manage women with DIP. All women managed in these maternity hospitals are seen in joined clinics by endocrinologists and obstetricians.

According to the national guidelines in Qatar, all pregnant women are offered screening for DIP. Women are classified as high risk if they have one of the following: a history of GDM, a history of pre-diabetes, a history of unexplained stillbirth, the birth of a baby with malformations associated with diabetes, a history of a macrosomic baby weighing ≥4 kg, pre-pregnancy BMI ≥25 kg/m, a history of polycystic ovary syndrome, and long-term steroid users. In the first trimester, all high-risk women not known to have pre-existing DM are screened with fasting plasma glucose and HBA1c, while others are screened by fasting plasma glucose. If the first test is normal, high-risk women are screened with a 75-g oral glucose tolerance test (OGTT) at 16–18 weeks gestation, and if normal, OGTT will be repeated at 24–28 weeks gestation, while low-risk women are screened at 24–28 weeks of gestation with 75 g OGTT. In the first trimester, GDM is diagnosed based on the following criteria: FBG 5.1–6.9 mmol or HBA1c 6.0%–6.4%. For OGTT, GDM is defined based on the WHO-2013 criteria: FBG 5.1–6.9 mmoL/l, 1 h ≥10.0 mmoL/l, and 2 h 8.5–11.0 mmol/L.^28^ Newly detected type 2 Diabetes mellitus (N-T2D) is diagnosed if the FBG ≥7.0 mmol/L, 2 h post-OGTT ≥11.1 mmol/L, or HBA1c ≥ 6.5%.^27^^,^^28^

All women with GDM are managed by nutritional therapy for 1–2 weeks.^29^ Women are asked to monitor their capillary blood glucose 4–7 times daily. The self-monitoring blood glucose (SMBG) targets are (FBG ≤5.3 mmol/L, 1-h post prandial ≤7.8 mmol/L or 2 h ≤ 6.7 mmol/L). Medical therapy starts if ≥ 20% of SMBG are above targets. According to Qatar national guidelines, Metformin is the first-line medical therapy unless it is contraindicated, unacceptable to the patient, or not tolerated.^30^ Insulin supplements metformin if the glucose targets are still not achieved or if metformin was not used for one of the above reasons. Women with normal glucose tolerance (NGT) do not routinely receive nutritional therapy.

### Data acquisition

Data was retrieved from Cerner, the electronic medical record. Pre-existing diabetes was defined as type 1 diabetes (T1D) or type 2 diabetes (T2D) diagnosed before the index pregnancy. To validate the data, we calculated the validation sample size using an online calculator (Australian Government, Australian Skills Quality Authority).^30^ At a 10% error level and 95% CI, we validated 100 charts.

### Outcomes

There were two primary outcomes: the screening rate and the prevalence of diabetes in pregnancy. We defined the screening rate as the proportion of women not known to have pre-existing DM who had any of the three tests: FBG, OGTT, or HBA1c. We calculated the prevalence of diabetes in pregnancy among those who were screened.

For secondary outcomes, we compared the demographics, including age and ethnicity; rates of live birth; rates of large for gestational age (LGA) (birth weight >90 percentile); small for gestational age (SGA) (birthweight <10th percentile, macrosomia (birth weight >4.0 kg), preterm delivery (delivery <37 weeks gestation), rates of C-section, and rates of Intra-uterine fetal death (IUFD) between women with GDM and those with no-GDM. We calculated the neonatal weight percentile using Fenton growth charts.^31^

### Statistics

We used Statistical STATA 15 software (College Station, TX: Stata Corp LP) for statistical analysis. We expressed categorical variables as numbers and percentages (%) and continuous variables as means (standard deviation). We defined the eligible population for screening as all women not known to have pre-pregnancy diabetes. We then excluded the unscreened women from the prevalence analysis. We expressed the prevalence of diabetes in percentage (%) with a 95% confidence interval (CI). Student t-test was used to compare continuous variables, while the age-adjusted odds ratio was used to compare categorical outcomes. p value < 0.05 was considered significant.

### Role of the funding source

The investigators received no funding for this study. The Medical Research Centre of the Academic Health System at Hamad Medical Corporation covered the publication fees.

## Results

**Fig. 1:**
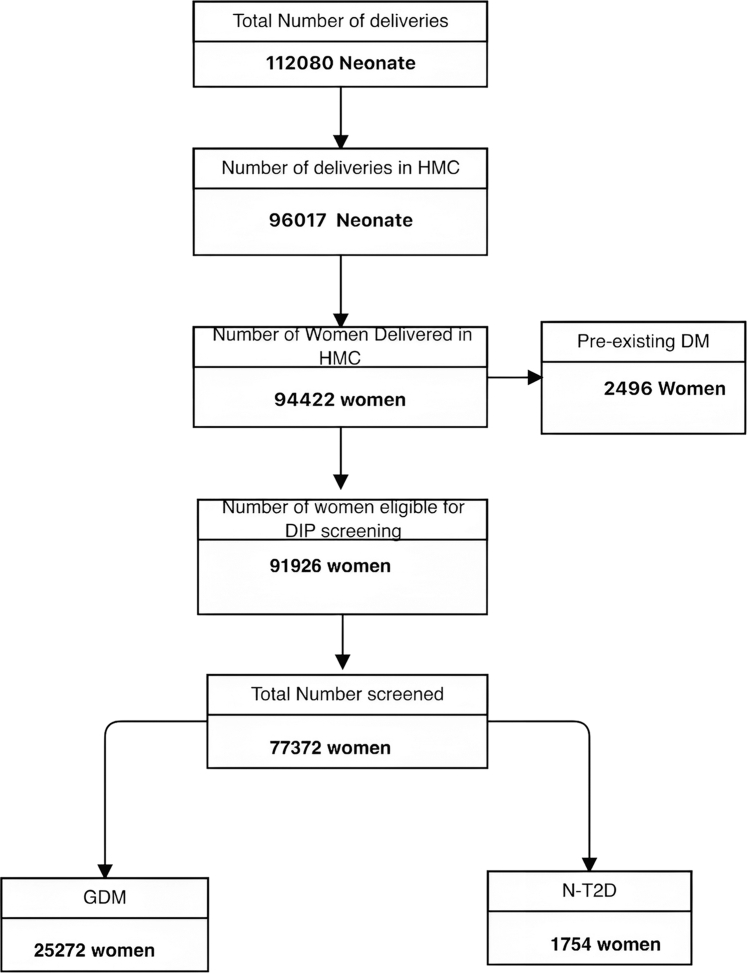
Study flow chart.

We included 94,422 women and 96,017 neonates (85.7%) out of 112,080 neonates born nationwide (Fig. 1). The mean age (SD) of the women was 30.0 (5.3) years, and 22,262 (23.6%) were Qatari. The number of women with pre-existing diabetes was 2496 women. As shown in Table 1, out of 91,926 women eligible for screening, 77,372 (84.2%) were screened for diabetes in pregnancy. Table 2 shows the prevalence of DIP among 79,868 women (women screened plus women with pre-existing diabetes). GDM was diagnosed in 25,272 women with a prevalence of 31.6% (95% CI: 31.3–32.0%). The prevalence of newly detected T2D (N-T2D) is 2.2% (95% CI: 2.1–2.3%). Pre-existing T2D and T1D prevalence was 2.9% (95% CI: 2.8–3.0%) and 0.2% (95% CI: 0.19–0.25), respectively.

**Table 1:** Baseline Characteristics and screening rates of 94,422.

Age, mean (SD)	30.0 (5.3) years	
Nationality (N/%)	Qatari	22,262 (23.6%)
	Arab	35,041 (37.1%)
	Asian	25,606 (27.1%)
	Others	11,513 (12.2%)
Number of deliveries/years	Y-2019	24,989 (26.5%)
	Y-2020	24,609 (26.0%)
	Y-2021	22,152 (23.5%)
	Y-2022	22,671 (24.0%)
Pre-existing Diabetes (% of deliveries)	2496 (2.7%)	
Eligible for screening (% of deliveries)	91,926 (97.4%)	
N of women screened (% of eligible)	77,372 (84.2%)	
Women with GDM (% of screened)	25,272 (32.6%)	
Women with N-T2D (% of screened)	1754 (2.3%)

**Table 2:** Prevalence of Diabetes in pregnancy among 79,868 women.

Diagnosis	Prevalence	95% CI
GDM	25,272 (31.6%)	31.3–32.0%
New T2D	1754 (2.2%)	2.1–2.3%
Pre-existing T2D	2322 (2.9%)	2.8–3.0%
Pre-existing T1D	174 (0.2%)	0.19–0.25%
Total of all DM	29,522 (36.9%)	36.6–37.3%

Table 3 compares the pregnancy outcomes between GDM (25,272 women) and non-GDM (50,346 women). Compared to the non-GDM group, women with GDM were significantly older (30.8 ± 5.3 versus 29.7 ± 5.2 years, p < 0.001). After adjusting for age, women with GDM had lower risk of IUFD (0.63 [95% CI 0.50–0.80, p < 0.001]) and SGA (0.88 [95% CI 0.84–0.92, p < 0.001]) but higher risk to deliver by C-section (OR 1.07 [95% CI 1.04–1.10, p < 0.001], and LFD (OR 1.09 [95% CI 1.01–1.15, p = 0.01]) compared to women with no GDM.

**Table 3:** Pregnancy outcomes in women with GDM and Non-GDM.

	GDM (n = 25,272)	Non-GDM (n = 50,346)	Un-adjusted OR (95% CI)	Unadjusted p-value	Adjusted OR^b^ (95% CI)	Adjusted p value
Age-years	30.8 ± 5.3	29.7 ± 5.2		0.001		
Mean neonatal weight (grams)	3166.5 ± 533	3155.3 ± 543		0.0068		
IUFD	91 (0.35%)	275 (0.54%)	0.66 (0.52–0.83)	0.001	0.63 (0.50–0.80)	<0.001
Preterm labour	2219 (8.8%)	4246 (8.4%)	1.04 (0.99–1.1)	0.09	1.0 (0.95–1.05)	0.90
C-section	9954 (39.4%)	18,177 (36.1%)	1.14 (1.11–1.19)	<0.001	1.07 (1.04–1.10)	<0.001
Macrosomia^a^	1232 (4.9%)	2334 (4.6%)	1.05 (0.98–1.13)	0.140	1.03 (0.96–1.11)	0.402
LGA^a^	1749 (6.8%)	3079 (6.0%)	1.12 (1.05–1.18)	<0.001	1.09 (1.01–1.15)	0.01
SGA^a^	7009 (13.7%)	3045 (11.9%)	0.86 (0.81–0.89)	<0.001	0.88 (0.84–0.92)	<0.001

The data are means + SD for age, number, and percentage in parentheses (%) for other categories.

a 0.44% of data are missing and not included in the data analysis.

b Odds Ratio adjusted for age.

## Discussion

To our knowledge, Qatar is the only country in the MENA (Middle East and North Africa) region to have a national DIP program. This program screened 84.2% of all eligible women between 2019 and 2022 inclusive. We have found that 36.9% of women have diabetes in pregnancy; 0.2% have pre-existing T1D, 2.9% have pre-existing T2D, 2.2% have newly detected T2D (N-T2D), and 31.6% have GDM. The pregnancy outcomes were slightly different between the GDM and non-GDM groups. The magnitude of these differences, despite statistical significance, is minimal. For example, the rates of IUFD were significantly lower in women with GDM compared to non-GDM; however, this translates to 2 fewer cases of IUFD per 1000 deliveries. The rates of LGA were lower in the non-GDM cases compared to GDM, but it translates to 8 fewer cases of LGA per 1000 deliveries.

Much of the debate regarding universal screening has revolved around the rise in the prevalence of GDM among low-risk patients.^32^ The shift from risk-based screening to universal screening in Finland increased the prevalence of GDM from 9.1% to 11.3%, with an increase in the proportion of diet-treated GDM.^10^ A study from Portugal showed that women with GDM and no risk factors have a lower risk of adverse pregnancy complications than those with GDM and risk factors.^11^ However, a study from Ireland showed that women with GDM detected on universal screening- and no risk factors were at higher risk of adverse pregnancy outcomes than those without GDM.^33^ A UK-based study reported reduced neonatal mortality and macrosomia after the shift to universal screening.^34^ Our study showed that universal screening increased the prevalence of GDM, and there were minor differences in adverse outcomes between those with and without GDM. Taken altogether, while universal screening increases the prevalence of GDM in lower-risk mothers, those women are still at risk of adverse pregnancy outcomes compared to women with no GDM and might still benefit from treatment.

Our study provides evidence for the instrumental role of primary care physicians in caring for women with diabetes during pregnancy. As outlined above, primary and secondary healthcare services use the same electronic medical records. Initial antenatal care is primarily provided in primary care. Screening for diabetes in pregnancy is embedded within the care plan for all pregnant women. Besides, the national program provides a clear pathway to escalate care from primary to secondary care. The screening rate in our study, 84.2%, was much higher than in other studies. Data from the UK, Ireland, and Tanzania reported screening rates of 63%, 44.0%, and 29.0% respectively.^34^, ^35^, ^36^ A study from Ireland showed that screening rates were significantly lower in primary care compared to secondary care, attributed to a lack of engagement by primary care physicians.^37^ Based on the previous study, the same group from Ireland suggested that screening for GDM in secondary care is more cost-effective than in primary care.^17^ However, our results argue that embedding the screening for GDM within primary care is crucial in increasing the uptake. Clear referral care pathways are another critical factor in supporting primary care physicians and improving their engagement.

Compared to previous studies from Qatar, the prevalence of GDM has remarkably increased with universal screening, from 16.9% to 21.5% in 2011 and 2016 to 31.6%.^25^^,^^26^ On the other hand, there was a reduction in the rates of maternal and fetal complications. The universal screening could explain the increase in the prevalence of GDM. However, the reduction of maternal and fetal complications reflects the broader changes in managing DIP through an all-encompassing framework that integrates primary care with obstetrics and diabetes care. At the secondary care level, all women with GDM are treated by a dedicated multi-disciplinary team comprised of Endocrinologists, Obstetricians, Diabetes educators, Dietitians, and Nurses within the maternity hospitals. An Endocrinologist and an Obstetrician jointly see all women throughout the pregnancy. This framework reduces the variability and unifies the care for all women with GDM in Qatar. We believe integrated systems are critical in providing care and reducing adverse pregnancy outcomes in women with GDM.

The study's main limitation is its retrospective nature, lack of data on critical adverse pregnancy outcomes such as pregnancy-induced hypertension and pre-eclampsia; no data on key risk factors such as BMI, gestational weight gain, parity, previous history of GDM, previous history of macrosomia and family history of diabetes; no data on other comorbidities such as polycystic ovary syndrome, essential hypertension, and dyslipidaemia. While the absence of this data does not impact the interpretation of the primary outcomes, it limits our ability to understand the factors that can predict low screening uptakes and those associated with increased risk of DIP. Furthermore, pregnancy outcomes are not corrected for critical known confounders such as age, ethnicity, BMI, gestational weight gain, socioeconomic status, parity, and smoking. For these reasons, care should be taken when interpreting these results. Finally, we could not capture screening tests outside the public health care system. There is a possibility that some women who are labelled as not screened were screened in private clinics.

The uptake rates for the universal screening for diabetes in pregnancy in Qatar are substantially higher than in other countries. Universal screening for diabetes in pregnancy is associated with a rise in the prevalence of GDM. Developing integrated care between primary and secondary care and embedding the screening for diabetes in pregnancy within primary care are essential for increasing screening uptake for DIP.

## Contributors

MB: - Conceptualisation, data analysis, data interpretation, data validation, writing the original draft. AA: - Conceptualisation, design, data interpretation, writing review, and editing. II, SB, HS, FT, K-A, MZ: - Data Interpretation, review of manuscript, and editing.

All authors have direct access to the data. Professor Abou-Samra reviewed the data and verified the results. All authors have full access to the data. The authors have read and approved the manuscript and have collectively decided to submit the study for publication.

## Data sharing statement

Data are available from the corresponding author upon reasonable request.

## Declaration of interests

The study did not receive any funds. The authors have no relevant affiliations or financial involvement with any organisation or entity with a financial interest in or conflict with the subject matter or materials discussed in the manuscript. This includes employment, consultancies, honoraria, stock ownership or options, expert testimony, grants, patents received or pending, or royalties.
